# Gut microbiota restructured by social isolation: Evidence for sex-specific response patterns

**DOI:** 10.1016/j.bbih.2026.101272

**Published:** 2026-05-25

**Authors:** Kathleen Fallon, Siva Reddy Challa, Casimir A. Fornal, Jerusha Boyineni, Marcelo Bento Soares, Sergey Malchenko, Krishna Kumar Veeravalli, Peter Gyarmati, Yajing Song

**Affiliations:** a Department of Biomedical Sciences, University of South Carolina School of Medicine Greenville, SC, USA; b Department of Cancer Biology and Pharmacology, University of Illinois College of Medicine Peoria, IL, USA; c Department of Neurosurgery, University of Illinois College of Medicine Peoria, IL, USA; d Department of Psychiatry and Behavioral Medicine, University of Illinois College of Medicine Peoria, IL, USA; e Department of Pediatrics, University of Illinois College of Medicine Peoria, IL, USA; f Department of Neurology, University of Illinois College of Medicine Peoria, IL, USA

**Keywords:** Post-weaning social isolation, Gut microbiota community structure, Sex-dependent response, Sex-specific response, Systems biology, Women's health

## Abstract

**Background:**

Both post-weaning social isolation and gut microbiota community structure have been independently associated with physical and mental health outcomes. However, sex differences are often overlooked in biology, medicine, and healthcare, leading to incomplete or biased research outcomes, medical interventions, and drug development. This study aimed to investigate the effects of post-weaning social isolation on gut microbiota community structure in young adult mice.

**Methods:**

A preclinical post-weaning social isolation mouse model was employed. Twenty-four 21-day-old C57BL/6 mice randomly assigned into social-isolated and co-housed groups [n = (6 males + 6 females)/group]. Social isolation started on postnatal day 22 and ended on postnatal day 78. Fecal samples were collected on postnatal days 22 before isolation (baseline) and 78 (endpoint) to investigate changes of gut microbiota diversity, composition, and the Firmicutes/Bacteroidetes ratio. The 16S metataxonomic sequencing data were analyzed using Mothur pipeline and R packages.

**Results:**

Our results indicated sex-related microbiota response patterns to post-weaning social isolation. All the significant changes in bacterial genera were observed exclusively in females, including *Akkermansia*, a sex hormone-responsive genus that supports butyrate production, *Oscillibacter*, a known butyrate producer, and *Parasutterella*, a proinflammatory and succinate-producing genus. The Firmicutes/Bacteroidetes ratio decreased in males mainly due to reduced Firmicutes and increased in females primarily due to reduced Bacteroidetes.

**Conclusion:**

Our findings highlight sex differences in gut microbiota responses to post-weaning social isolation, with particularly pronounced effects in females. These results provide a foundation for exploring how the gut microbiota integrates social environmental signals to influence health outcomes. As a preclinical, observational study, our findings may suggest preliminary insights for future translational research aimed at understanding women's health.

## Highlights

• Sex is s a critical biological variable in the microbiome studies.
• Young females exhibit stronger microbiota responses to post-weaning social isolation than males.
• Social isolation induces broader taxonomic alterations in young females, with multiple phyla and genera affected.
• Social isolation induces more limited microbiota alterations in young males.
• Socially isolated young females show greater relative weight gain than males.

## 1 Introduction

The term ‘sex’ in this article refers to the biological characteristics that differentiate males and females. Sex differences arise from genetic and hormonal mechanisms, potentially interacting with social environmental conditions such as social isolation (SI) (Szadvari et al., 2022). For example, social stress can modulate the expression of genes related to hormones, their receptors, and associated enzymes through epigenetic mechanisms (Shepard et al., 2009). These differences impact nearly every aspect of biological processes through multiple pathways and physiological systems. For instance, both innate and adaptive immune responses are generally more robust in females than in males (Klein and Flanagan, 2016), leading to faster pathogen clearance and higher antibody titers following vaccination (Klein and Flanagan, 2016; Gal-Oz and Shay, 2022). However, this heightened immune responsiveness is also associated with a greater prevalence of autoimmune diseases among females (e.g., systemic lupus erythematosus) (Klein and Flanagan, 2016; van Drongelen et al., 2025). Additionally, the hypothalamic-pituitary-adrenal (HPA) axis exhibits a stronger stress responsiveness and impaired negative feedback in females, which have been associated with depression and anxiety (Young, 1998). From an evolutionary perspective, it has been proposed that the substantial energetic demands associated with female reproduction may have enhanced mitochondrial function and favored greater energy storage capacity compared to males (Mauvais-Jarvis, 2024). Maternal inheritance of mitochondria has been associated with sex differences in metabolism regulation and disease risk, including obesity prevalence (van Drongelen et al., 2025; Saben et al., 2016). Given these diverse and systemic effects, it is essential to account for sex-related differences in study design, analysis, and reporting to ensure accurate and generalizable results for both males and females (Mogil, 2016; Rechlin et al., 2022).

SI is increasingly recognized as a significant public health concern due to its association with adverse health outcomes (Holt-Lunstad, 2021; Wang et al., 2023). Exposure to SI during critical neurodevelopmental periods, including childhood and adolescence, can produce lasting influence on brain mature, stress responsiveness, and social emotional functioning in both humans (Corrigan et al., 2024; Dragan et al., 2019; Koss and Gunnar, 2018) and animals (Myers, 2024; Noback et al., 2021; Fontenot et al., 2018; Graf et al., 2024; Challa et al., 2023). In humans, adversity during childhood and adolescence has been linked to alterations in gray matter volume, cognitive development, and HPA axis programming, as well as persistent socioemotional consequences. Similarly, in animal models, post-weaning SI affects social behavior and neuro-modulatory systems. Post-weaning SI is widely used as a neurodevelopmental preclinical model with replicable behavioral and neural consequences (Fone and Porkess, 2008; Lukkes et al., 2009; Walker et al., 2019).

The gut microbiota – comprising trillions of microorganisms, primarily bacteria – resides in the intestinal tract of humans and animals. This microbiota ecosystem co-evolves with its host and contributes to the development and regulation of key physiological systems, such as the immune (Ley et al., 2008) and central nervous systems, among others (Cryan et al., 2019). It plays a crucial role in maintaining overall health throughout the lifespan (Ley et al., 2008; Cryan et al., 2019; Gabaldón, 2021). Notably, the composition of the gut microbiota differs by sex in both humans and animals, even when controlling for other influencing factors such as age, environment, and lifestyle (Kim et al., 2020).

However, the sex-related effects of SI during childhood and adolescence on the gut microbiota remain largely underexplored, with limited insights from animal studies (Hurst et al., 2025; Lopizzo et al., 2021) and no investigations in humans (Agusti et al., 2023). We hypothesize that post-weaning SI influences development and physiological homeostasis via sex-dependent alterations in the gut microbiota and its metabolites. Accordingly, we aimed to investigate whether it differently affects the gut microbiota community structure in male and female young mice (postnatal day [PND] 22-78) relative to co-housing (CH) controls.

## 2 Methods

### 2.1 Animal housing and experimental design

**Fig. 1:**
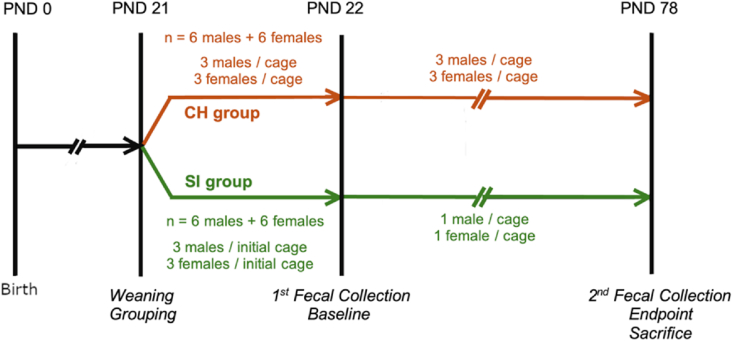
Experiment timelines. Colors denote different groups (CH: brown, SI: green). Abbreviation: PND, postnatal day. CH, co-housing. SI, social isolation. (For interpretation of the references to color in this figure legend, the reader is referred to the Web version of this article.)

As in our previous study (Song et al., 2025), C57BL/6 mice were obtained from the Jackson Laboratory (Bar Harbor, ME). All experimental animals were second-generation offspring of C57BL/6 mice. As they were bred and maintained in the University of Illinois College of Medicine Peoria Animal Facility, no quarantine or acclimatization period was required. At PND 21, mice were randomly housed in conventional cages and allocated to CH or SI groups (n = 12 mice/group, (6 males + 6 females)/group, 3 same sex/initial cage). Following the Animal Research: Reporting of in Vivo Experiments (ARRIVE) guidelines, all procedures were conducted under the same conditions, including controlled temperature (21 ± 1 °C), a 12:12-h light/dark cycle, and ad libitum access to regular chow and water (Purina LabDiet 5). Handling and cage changes were performed consistently by the same trained personnel. As part of the socially isolated condition, environmental enrichment was withheld from the SI group but made available to the CH group. Mice in the CH and SI groups were housed in two separate, quiet, controlled-access rooms. After an initial 24-h CH period, SI mice were subsequently housed individually with opaque partitions placed between each pair of cages to prevent visual contact. The CH and SI conditions were maintained for 56 days (Challa et al., 2023; Song et al., 2025). Fresh fecal pellets (12 samples/time/group, 6 samples/sex/time/group) were collected on PND 22 (“before” or baseline, prior to isolation) and PND 78 (“after” or endpoint) (Fig. 1). Body weight was measured on PNDs 22, 50, and 77 or 78 (Challa et al., 2023). At the end of the study, all mice were euthanized with an overdose of pentobarbital (150 mg/kg; i.p.) followed by transcardial perfusion with 1×PBS (Fig. 1). The experimental protocol was approved by the University of Illinois College of Medicine Peoria Institutional Animal Care and Use Committee (IACUC) review board (protocol number 1843072) and was conducted in accordance with institutional guidelines and the ARRIVE guidelines.

### 2.2 16S rRNA gene sequencing

Following the manufacturer's instructions, fecal DNA was extracted using the QIAamp DNA Stool Mini Kit (Qiagen) (Song et al., 2022) on the same day as collection and stored at −20 °C until sample delivery. Universal primers (515F and 806R) were used to amplify the V4 hypervariable region of the 16S rRNA gene for bacterial community profiling. The amplicon library was prepared and sequenced at the Roy J. Carver Biotechnology Center (University of Illinois at Urbana-Champaign). Briefly, PCR products were purified, indexed, normalized, and pooled. The pooled library was sequenced using paired-end reads (2×250 base pairs) on an Illumina Miseq platform with a MiSeq Reagent Kit v2 (500-cycle) (Illumina). A 5% PhiX control library was spiked into the sequencing run to increase sequence diversity and improve base-calling accuracy for a low-diversity amplicon library. DNA extraction reagents and a blank template were included as negative controls to monitor contamination.

FASTQ files were generated and demultiplexed using the bcl2fastq v2.20 Conversion Software (Illumina). Primer and adaptor sequences were trimmed by the Roy J. Carver Biotechnology Center. Amplicon sequencing of the V4 region of the bacterial 16S rRNA gene yielded total 1,076,982 reads (mean ± standard deviation [*SD*] per sample: 11,167 ± 2,782).

### 2.3 Data analysis

Metataxonomic raw reads were filtered with Phred quality scores ≥ Q30 (FLASH (Magoč and Salzberg, 2011)). The resulting sequences were assembled and processed using Mothur v.1.48.0 (Schloss et al., 2009) via the Windows command line environment following the Miseq standard operating procedure (Kozich et al., 2013). Briefly, the pipeline includes removal of ambiguous bases, abnormal lengths, and duplicate sequences, alignment to reference database, pre-clustering, and chimera removal. Operational taxonomic unit (OTU) were clustered at 97% similarity to describe and compare microbiota communities, and taxonomic classification was conducted using the SILVA v138 reference database. After clustering sequences into OTUs, data were normalized to standardize sequencing depth across samples (Schloss et al., 2009; Kozich et al., 2013). Microbiota α-diversity (within-sample diversity) was evaluated using the inverse Simpson index, which quantifies both the number and relative abundance of taxa, provides more weight to abundant taxa, and is more stable across sequencing depths. Higher α-diversity indicates a more diverse and evenly distributed microbiota community within individual samples (Finotello et al., 2018; Xia and Sun, 2023). Microbiota β-diversity (between-sample diversity) distance matrices were calculated using Bray-Curtis dissimilarity to evaluate differences in community composition and abundance since Bray-Curtis performs well when differences are abundance-driven without requiring phylogenetic tree reconstruction and presence/absence issues. In Principal Coordinate Analysis (PCoA) plots, clustering reflects similarity in microbiota composition, whereas dispersion indicates increased variability (Finotello et al., 2018; Borman et al., 2026). Visualization of gut microbiota β-diversity and taxonomic composition based on OUT-based methods were performed in R (Wickham et al., 2019; Wickham, 2016) (v. 4.2.2) using Tidyverse and ggplot2 packages. The figures were assembled and formatted using GIMP (v 2.10). The Firmicutes and Bacteroidetes ratio (F/B ratio) was calculated for each mouse using relative abundance values (Firmicutes relative abundance divided by Bacteroidetes relative abundance) at baseline [F/B _(before)_] or endpoint [F/B_(after)_]. The fold-change was measured as F/B _(after)_ divided by F/B _(before)._

Statistical analyses were performed using the Mann-Whitney *U* test (for comparisons between two independent groups) and Wilcoxon Signed-Rank test (for repeated measures within the same group) (Paliy and Shankar, 2016). Analysis of Molecular Variance (AMOVA) was applied to evaluate the effects of between-group and within-group variations. P-values in multiple hypothesis testing were adjusted using the Benjamini-Hochberg (BH) correction (*p.adj*). A *p*-value (single comparison) or *p.adj*-value (multiple comparisons) < 0.05 was considered statistically significant. Data were presented as mean ± *SD* or median with interquartile range (*IQR*), as appropriate.

## 3 Results

Overall, the results showed sex-related effects, including both sex-dependent differences between males and females and sex-specific alterations observed in one sex.

### 3.1 Sex-dependent differences in the Firmicutes/Bacteroidetes ratio

**Fig. 2:**
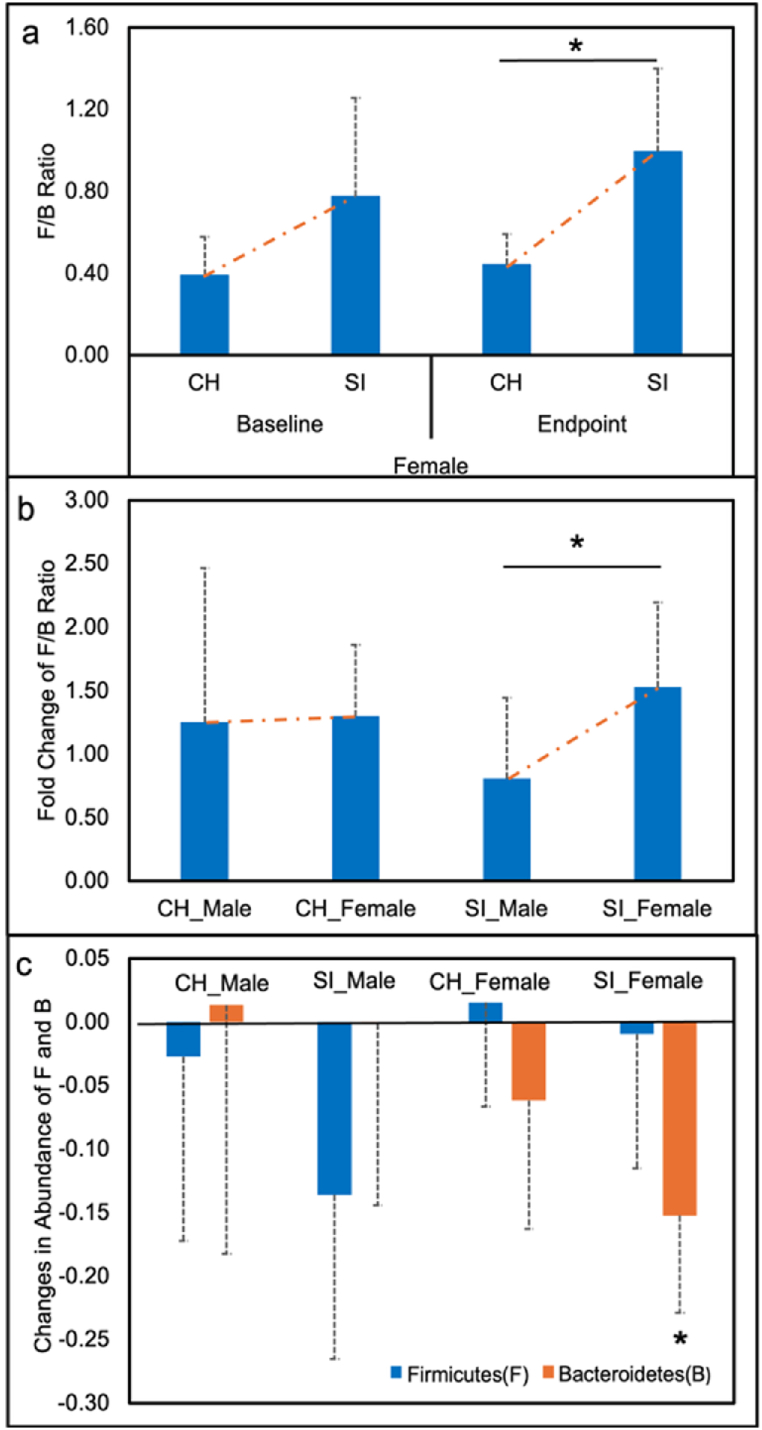
Alterations in Firmicutes (F) and Bacteroidetes (B) in males and females under CH and SI conditions. (a) Cross-sectional F/B ratio in females under different housing conditions. (b) Longitudinal changes in the F/B ratio by sex under each housing condition. (c) Relative abundance changes in Firmicutes and Bacteroidetes. ΔF (blue) and ΔB (orange), calculated as ‘after – before’. Error bars represent standard deviation (*SD*). Abbreviation: CH, co-housing. SI, social isolation. “∗” indicates *p* < 0.05. (For interpretation of the references to color in this figure legend, the reader is referred to the Web version of this article.)

We analyzed cross-sectional differences in Firmicutes and Bacteroidetes relative abundance and F/B ratio [(F/B _(before)_ and F/B _(after)_] by housing condition and by sex, as well as longitudinal changes expressed as fold change over time [F/B _(after)_ ÷ F/B _(before)_]. At the endpoint, a housing-dependent difference was observed only in females, with SI mice exhibiting a lower Bacteroidetes relative abundance (CH: 0.59 ± 0.07, SI: 0.39± 0.08, *p* = 0.004) and a higher F/B ratio than CH (CH: 0.44 ± 0.15, SI: 0.99± 0.40, *p* = 0.002) (Fig. 2a). No significant differences were detected at baseline. The corresponding differences in Firmicutes did not significantly differ between baseline and endpoint. Longitudinal changes in the F/B ratio differed significantly between sexes under SI (*p* = 0.03) but not under CH (Fig. 2b). SI males display a decrease in F/B ratio over time (0.81 ± 0.64), whereas SI females exhibited an increase (1.53± 0.67). These opposite trajectories were primarily driven by reduced Firmicutes in males ((ΔF = −0.14 ± 0.13, *p* = 0.06, *d* = 0.76) and reduced Bacteroidetes in females (ΔB = −0.15 ± 0.08, *p* = 0.03, *d* = 0.88) (Fig. 2c).

### 3.2 Sex-specific differences in relative weight gain (%)

Relative weight gain (%) exhibited a sex-specific response to housing conditions. Females under SI gained significantly more weight than those under CH at PNDs 50 (CH: 46.53 ± 14.88, SI: 88.28± 16.51, *p* = 0.002) and 78 (CH: 63.78 ± 19.06, SI: 105.18± 15.85, *p* = 0.004), whereas no significant differences were observed between CH and SI in males at either PND (Challa et al., 2023) (Suppl. Fig. 1).

### 3.3 Sex-dependent differences in the gut microbiota diversity

**Fig. 3:**
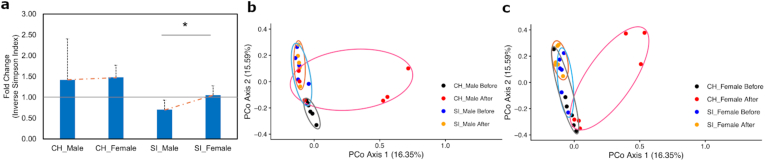
Changes in α-diversity (a) and β-diversity before and after CH and SI in male (b) and female mice (c). (a) A significant change in α-diversity fold change is observed only under SI, with a reduction in males and no change in females. Error bars represent standard deviation (*SD*). (b-c) β-diversity shows dispersion after CH and clustering after SI in both males and females. Black and blue circles represent gut microbiota distribution before CH and SI, respectively, while rose pink and light brown circles represent distribution after CH and SI. The color scheme is not associated with sex. Each dot represents one mouse. Abbreviation: CH, co-housing. SI, social isolation. “∗” indicates *p* < 0.05. (For interpretation of the references to color in this figure legend, the reader is referred to the Web version of this article.)

The fold change (after ÷ before) in α-diversity under SI differed significantly between male and female mice (*p* = 0.026) rather than CH (Fig. 3a). Both males and females under CH or SI showed temporal changes in β-diversity, with CH mice exhibiting greater dispersion and SI mice displaying more tightly clustering (1 < *Fs* < 2, Fig. 3b and c). CH_males differed significantly (*p.adj* = 0.029), but not in SI_females (*p.adj* = 0.058) and SI_males (*p.adj* = 0.232). The corresponding Δβ-diversity values were not significantly different.

### 3.4 Sex-dependent differences in the gut microbial composition

After analyzing the complete dataset, first, we assessed sex-related differences in gut microbiota composition by focusing on abundant taxa, including all observed taxa at the phylum level (Suppl. Fig. 2) and the top 10 most abundant taxa at the genus level (Suppl. Fig. 3). A total of six taxonomically classified phyla were detected in all mice, with sex-dependent alterations. Under CH, four phyla (Bacteroidetes, Firmicutes, Tenericutes, and Actinobacteria) exhibited opposite trends between males (Suppl. Fig. 2a) and females (Suppl. Fig. 2b). Under SI, the average abundance of Bacteroidetes and Actinobacteria remained unchanged in males (Suppl. Fig. 2c), whereas a decrease in Bacteroidetes and an increase in Actinobacteria were observed in females (Suppl. Fig. 2d). The changed trends in abundance of Proteobacteria and Verrucomicrobia were consistent across sexes under both patterns. Among the top 10 taxa observed at the genus level, six were taxonomically assigned at the genus level. One (*Anaeroplasma*) exhibited an opposite trend between sexes under CH (Suppl. Fig. 3a and 3b) but not under SI (Suppl. Fig. 3c and 3d), and the remaining five genera followed similar trends in both sexes under both patterns.

**Fig. 4:**
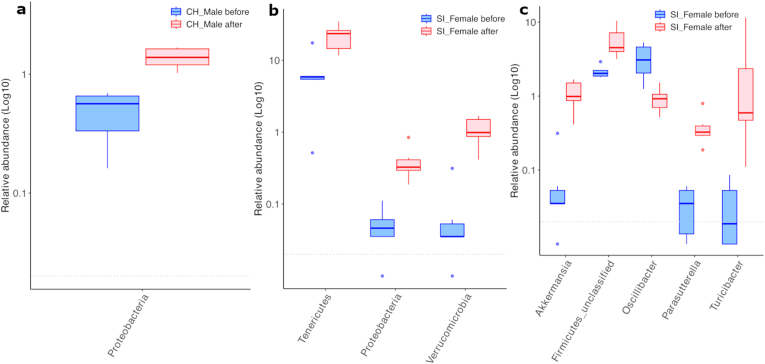
Sex-dependent differences in gut microbiota composition under CH and SI conditions. (a) Significantly different phylum in CH males. (b) Significantly different phyla in SI females. (c) Significantly different taxa at the genus level (four classified, one unclassified) in SI females. Error bars represent interquartile range (*IQR*) across all panels. Abbreviation: CH, co-housing. SI, social isolation. Significance indicates adjusted *p*-value <0.05.

Next, we assessed significantly longitudinal shifts in gut microbiota composition by sex under either CH or SI (*p.adj* < 0.05). Only one significant phylum was observed in the CH group — Proteobacteria, which was present in males but not in females (Fig. 4a). In contrast, all significant changes at both phylum (Tenericutes, Proteobacteria, and Verrucomicrobia) and genus levels (*Akkermansia*, *Turicibacter, Parasutterella*, *and Oscillibacter*) within the SI group were observed exclusively in females (Fig. 4b and c).

## 4 Discussion

Our previous work demonstrated that social interaction patterns influence gut microbiota community structure in mice (Song et al., 2025). CH increased microbiota diversity and stabilized community composition, likely through physical contact, coprophagic behavior, shared environment, and multiple-cage effects. In contrast, SI reduced microbiota diversity and was associated with decreased ecological stability of the microbiota community, presumably due to diminished horizontal microbiota exchange among social partners (Song et al., 2025; Briggs et al., 2025). Here, we further examined sex-related differences and found that post-weaning SI in mice reshaped the gut microbiota community differently in males and females, including α-diversity, β-diversity, F/B ratio, and composition at both the phylum and genus levels, with more pronounced changes observed in females.

Findings in previous studies partially align with our results, but with important limitations compared to the current work. One study observed similar trends in both α- and β-diversity but the analysis was restricted to male rats (Dunphy-Doherty, 2018). A separate study in mice concluded that post-weaning stress responses in both α- and β-diversity were sex-dependent and primarily affected male mice (Yin et al., 2024). Our study demonstrated sex-dependent responses to post-weaning SI in α-diversity, with a reduction observed in males and relative stability in females. Additionally, another study reported significant female-specific differences in β-diversity between SI and CH conditions, driven by between-group variability (Lopizzo et al., 2021). In our study, we observed a similar trend toward greater between-group variation in SI females compared to SI males; however, this did not reach statistical significance, suggesting a potential effect that may require further investigation with larger sample sizes. Our results also show that β-diversity in males was more sensitive to CH than females. These changes suggest a broader effect of sex under different social environmental exposures on microbiota structure. Finally, although a human study reported decreased α-diversity following childhood stress, sex differences were not conducted (Nathalie et al., 2019), underscoring the need for sex-resolved analysis—an aspect directly addressed in our work.

Firmicutes and Bacteroidetes, the two most abundant bacterial phyla in the feces of both humans and mice (Ley et al., 2005), have been reported to participate in energy metabolism and fat storage (Macfarlane and Macfarlane, 2003; Jumpertz et al., 2011). Thus, F/B ratio has been used as one of the parameters to identify altered metabolic regulations, particularly the pathophysiology of obesity caused by dysbiosis in both humans and mice (Ley et al., 2005, 2006; Turnbaugh et al., 2006). A higher F/B ratio has been proposed to associate with greater weight gain and adiposity. In contrast, a lower ratio has been linked to reduced weight gain (Ley et al., 2005; Turnbaugh et al., 2006) or weight loss (Ley et al., 2006). However, these associations were inconsistent, particularly in humans (Sze and Schloss, 2016), and these studies did not account for sex effects.

Our findings highlight the importance of accounting for sex in microbiota analyses. In the absence of analysis by sex, both post-weaning CH and SI conditions did not significantly affect the F/B ratio and weight gain (Song et al., 2025). By contrast, sex-stratified analysis showed that the endpoint significant increase in F/B ratio and the significant reduction in Bacteroidetes occurred only in SI females, not in CH females. Baseline levels of Bacteroidetes and the F/B ratio did not differ between housing conditions, indicating that these changes likely reflect post-isolation effects rather than pre-existing differences. Longitudinally, Firmicutes and Bacteroidetes responded to SI in a sex-dependent manner, with a reduction in Firmicutes in males and a decrease in Bacteroidetes in females, contributing to divergent F/B ratio trajectories between sexes. These alterations in the two dominant bacterial phyla were accompanied by greater weight gain in females and less weight gain in males over time. Previous studies (Macfarlane and Macfarlane, 2003; Jumpertz et al., 2011) have linked higher Firmicutes and lower Bacteroidetes to increase energy harvest and fat deposition but have not accounted for sex differences. Collectively, our findings suggest that sex-dependent remodeling of F/B ratio under SI may be linked to differences in weight variation. However, as this ratio represents a coarse index of community structure, these interpretations remain tentative and require validation through functional and species-level profiling.

A study has reported that lack of social contact during adolescence produced sex-dependent gut microbiota alterations in rats, for example, *Akkermansia* increased at PND 49 in females but not in males (Lopizzo et al., 2021), and SI led to more pronounced alterations in gut microbiota in female rats, including the reduced *Oscillibacter* (Hurst et al., 2025). Consistent with these earlier findings, our data suggest sex-dependent differences in the gut microbiota composition under both CH and SI conditions, particularly in females subjected to post-weaning SI. These shifts were characterized by significant increases in the relative abundances of Tenericutes, Proteobacteria, and Verrucomicrobia at the phylum level, as well as significant increases in *Akkermansia*, *Turicibacter*, and *Parasutterella*, alongside a significant decrease in *Oscillibacter* at the genus level.

The genus *Akkermansia* (phylum Verrucomicrobia) comprises mucin-degrading bacteria increasingly recognized as modulators of gut, immune, and metabolic physiology (Panzetta and Valdivia, 2024). Numerous studies associate higher *Akkermansia* abundance with improved health outcomes (Yu et al., 2023; Bae et al., 2022; Yoon et al., 2021). However, a growing body of evidence indicates that beneficial effects are highly context-dependent and influenced by multiple internal and external factors, including diet (Wolter et al., 2024), immunometabolism status (Panzetta and Valdivia, 2024), and gut microbiota ecology (Chia et al., 2018). Importantly, emerging evidence shows that *Akkermansia* is estrogen-responsive (Sakamuri et al., 2023; Acharya et al., 2022; Peters et al., 2022) and may play a key role in female health. For example, rodent studies indicated that gonadectomy reduced *Akkermansia* abundance compared to gonad-intact females and β − estradiol promoted *Akkermansia* growth (Sakamuri et al., 2023). Human studies of the menopausal transition likewise demonstrated that reduced *Akkermansia* was associated with declining estrogen levels after adjusting covariates (age, demographics, behavior) (Peters et al., 2022). Additionally, *Akkermansia* has been associated with behavioral phenotype in rodent models, with increases linked to depression-like behavior in postpartum mice (Xu et al., 2024), whereas in male mice elevated abundance has been linked to improved behavior outcome (Ding et al., 2021). Our previous study using the same post-weaning SI model showed mild depression-like behavior in females but not in males (Challa et al., 2023). In this study, we observed a sex-specific *Akkermansia* response to SI: females exhibited a pronounced increase in *Akkermansia* abundance (Fig. 4c). These parallel sex-specific patterns observed across studies may suggest that responses of *Akkermansia* are condition- and sex-dependent.

*Turicibacter* and *Parasutterella*, genera within the phyla Firmicutes and Proteobacteria, respectively, have been associated with immune responses, systemic metabolic regulation, and body weight changes in prior studies (Lynch et al., 2023; Gao et al., 2020; Hidalgo-Garcia et al., 2023; Ying et al., 2022; Gu et al., 2019; Ju et al., 2019). Notably, *Turicibacter* exhibited sex-dependent effects on both immune and metabolic pathways: it promotes anti-inflammatory responses in male mice (Gao et al., 2020), while it enhanced pro-inflammatory responses and potentially disrupted metabolic homeostasis in female mice (Lynch et al., 2023; Hidalgo-Garcia et al., 2023). In contrast, although previous studies found that *Parasutterella* was correlated with the regulation of energy metabolism and intestinal inflammation in animal models*,* sex-induced differences have not been systematically investigated (Ying et al., 2022; Gu et al., 2019; Ju et al., 2019). Partly consistent with these reports, our study found increased *Turicibacter and Parasutterella* and weight gain in females but not in males; however, further studies on immune responses and metabolic regulation are required.

Short-chain fatty acids (SCFAs), which are key gut microbiota-derived metabolites, play a crucial role in maintaining barrier integrity to maintain homeostasis of intestine and the central nervous system (Peng et al., 2009; Kelly et al., 2015; Braniste et al., 2014; Hoyles et al., 2018). This is achieved through cross-feeding interactions between SCFA-producing bacteria (Fusco et al., 2023) and their metabolic helpers (Paone and Cani, 2020). In this study, female mice exposed to SI exhibited a significant decrease in the relative abundance of *Oscillibacter*, a genus of butyrate-producing Firmicutes, alongside an increase in *Akkermansia*, a known helper in butyrate production (Belzer et al., 2017) (Fig. 4c). Given the recognized roles of butyrate-producing gut bacteria in maintaining barrier integrity, further studies are warranted to investigate how microbiota alterations relate to physiological functions.

This study provides an initial characterization of sex-related differences in the gut microbiota in response to post-weaning SI. Several limitations should be addressed in future research. Large sample size should be used to confirm and extend the present findings. Reversal experiments, such as resocialization or dietary interventions, should be conducted to assess the potential for reversing gut dysbiosis-induced pathophysiology by age and sex. Studies of sex differences are needed to clarify the potential mechanistic links between the F/B ratio and energy metabolism, bacteria-derived metabolites and gut permeability, as well as behavioral outcomes. Additionally, monitoring the estrous cycle will be important to determine whether hormonal fluctuations contribute to the observed female-specific microbiota patterns.

Although the post-weaning SI mouse model is well-established for studying disease mechanisms, further human studies are required to account for species-specific differences. These should include observational studies in children and/or adolescence, as well as longitudinal and interventional studies to establish the translational relevance of sex-related differences. Shotgun metagenomics will help achieve this by improving taxonomic resolution and characterizing functional alterations.

## 5 Conclusion

Post-weaning social interaction patterns play distinct roles in shaping the gut microbiota community structure, with particularly notable effects in females exposed to SI compared to both CH females and SI males. These findings extend our previous report linking SI to gut dysbiosis (Song et al., 2025) and emphasize the importance of sex as a biological variable in microbiome studies (Kim et al., 2020). Our findings may provide a foundation for future studies exploring sex-related microbiota response patterns to social environmental exposure within a systems biology framework. Such integrative approaches may help elucidate how microbiota relate to host physiology regulation and, over time, inform microbiota-based precision medicine strategies for women health.

## Consent to publish declaration

Not applicable.

## Ethics approval and consent to participate declarations

All animal experiments were approved by the Institutional Animal Care and Use Committee (IACUC) of the University of Illinois College of Medicine at Peoria (protocol number 1843072).

## Funding

This research received no specific grant from any public, commercial, or non-profit funding agency.

## CRediT authorship contribution statement

**Kathleen Fallon:** Writing – review & editing. **Siva Reddy Challa:** Methodology, Writing – review & editing. **Casimir A. Fornal:** Methodology, Writing – review & editing. **Jerusha Boyineni:** Methodology, Writing – review & editing. **Marcelo Bento Soares:** Resources, Writing – review & editing. **Sergey Malchenko:** Writing – review & editing. **Krishna Kumar Veeravalli:** Methodology, Writing – review & editing. **Peter Gyarmati:** Data curation, Formal analysis, Methodology, Resources, Software, Validation, Writing – review & editing. **Yajing Song:** Conceptualization, Data curation, Formal analysis, Investigation, Methodology, Project administration, Resources, Software, Validation, Visualization, Writing – original draft, Writing – review & editing.

## Declaration of competing interest

None.

## Data Availability

The raw data generated in this study have been deposited in the NCBI BioProject ID PRJNA1011329.
