# Supplementary material for: Gut microbiota restructured by social isolation: Evidence for sex-specific response patterns
Source: Brain Behav Immun Health. 2026 May 25;54:101272. doi: 10.1016/j.bbih.2026.101272 (PMC13235384; doi:10.1016/j.bbih.2026.101272)
Supplement: Multimedia component 1 [file mmc1.pdf]

Gut Microbiota Restructured by Social Isolation: Evidence for Sex-Specific Response Patterns

Kathleen Fallon[a], Siva Reddy Challa[b], Casimir A Fornal[b], Jerusha Boyineni[b], Marcelo Bento Soares[b,c,d], Sergey Malchenko[b], Krishna Kumar Veeravalli[b,c,e,f], Peter Gyarmati[a], Yajing Song[a] *

[a] Department of Biomedical Sciences, University of South Carolina School of Medicine Greenville, SC, USA
[b] Department of Cancer Biology and Pharmacology, University of Illinois College of Medicine Peoria, IL, USA
[c] Department of Neurosurgery, University of Illinois College of Medicine Peoria, IL, USA
[d] Department of Psychiatry and Behavioral Medicine, University of Illinois College of Medicine Peoria, IL, USA
[e] Department of Pediatrics, University of Illinois College of Medicine Peoria, IL, USA
[f] Department of Neurology, University of Illinois College of Medicine Peoria, IL, USA

*** Corresponding author: yajing@greenvillemed.sc.edu**

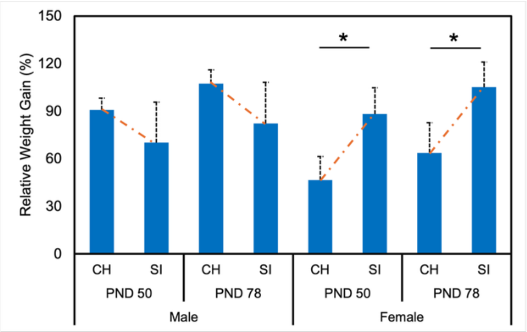

Supple. Fig. 1 Sex differences in relative weight gain (%), as previously reported [1]. Error bars represent standard deviation (*SD*). "*" indicates $p < 0.05$. Abbreviation: CH, co-housing. SI, social isolation. PND, postnatal day.

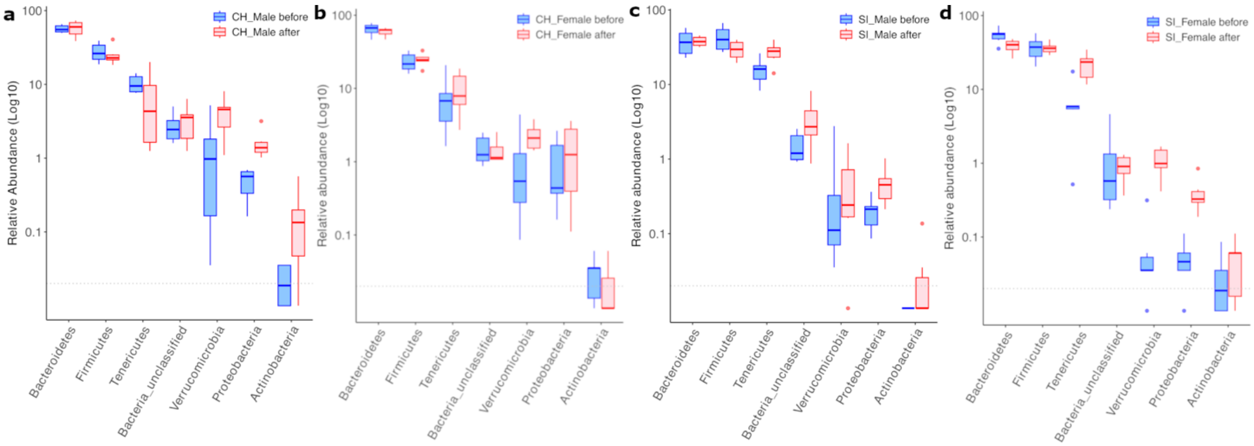

Suppl. Fig. 2 Relative abundances of taxa at the phylum level before and after CH or SI in males and females: (a) CH males, (b) CH females, (c) SI males, and (d) SI females. Abbreviation: CH, co-housing. SI, social isolation.

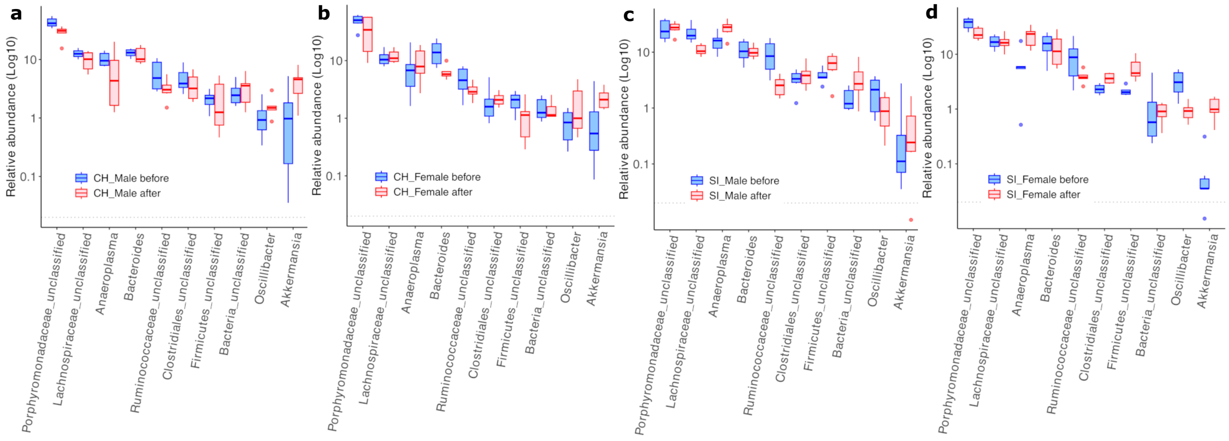

Suppl. Fig. 3 Relative abundances of the top 10 taxa at the genus level before and after CH or SI in males and females: (a) CH males, (b) CH females, (c) SI males, and (d) SI females. Abbreviation: CH, co-housing. SI, social isolation.

Reference

1. Challa SR et al. 2023. The impact of social isolation and environment deprivation on blood pressure and depression-like behavior in young male and female mice. Chronic Stress (Thousand Oaks).7:24705470231207010.
